# Dynamic tuning of Bloch modes in anisotropic phonon polaritonic crystals

**DOI:** 10.1038/s41377-025-02157-6

**Published:** 2026-01-03

**Authors:** Junbo Xu, Ke Yu, Xiang Ni, Enrico M. Renzi, Lei Zhou, Yanzhen Yin, Zhou Zhou, Zhichen Zhao, Tao He, Di Huang, Kyoung-Duck Park, Zhanshan Wang, Andrea Alù, Tao Jiang

**Affiliations:** 1 MOE Key Laboratory of Advanced Micro-Structured Materials, Shanghai Frontiers Science Center of Digital Optics, Institute of Precision Optical Engineering, and School of Physics Science and Engineering, Tongji University, Shanghai, China; 2 School of Physics, Central South University, Changsha, Hunan China; 3 Photonics Initiative, Advanced Science Research Center, City University of New York, New York, NY USA; 4 Physics Program, The Graduate Center, City University of New York, New York, NY USA; 5 Department of Physics and Department of Semiconductor Engineering, Pohang University of Science and Technology (POSTECH), Pohang, Republic of Korea; 6 Shanghai Institute of Intelligent Science and Technology, Tongji University, Shanghai, China

**Keywords:** Polaritons, Sub-wavelength optics

## Abstract

Phonon polaritons, arising from the coupling of photons with lattice vibrations, enable light confinement on deeply subwavelength scales. Phonon polaritonic crystals (PoCs), leveraging these inherently low-dissipation excitations, have further shown exceptional potential for nanoscale light manipulation through engineered Bloch modes. Yet, their static nature has so far hindered dynamic modulation, thus limiting their adaptability for real-time applications. Here, we demonstrate in situ electrostatic control of low-loss anisotropic phonon-polaritonic Bloch modes in α-MoO_3_ patterned into a periodic hole array with a graphene gate. Through theoretical calculation and real-space nano-imaging, we show that electrostatic gating dynamically modulates key characteristics of Bloch modes in hybrid α-MoO_3_/graphene PoCs. Critically, gating reshapes the PoC band structure, spectrally aligning high-density-of-states flat-band regions with the excitation laser frequency, thereby selectively amplifying Bloch mode resonances. We further achieve on-demand switching over far-field leakage of Bloch modes by electrostatically steering these flat bands across the light cone. Our work establishes a platform for adaptive nanostructured phonon polaritonic devices. This advancement not only facilitates directional control of low-loss anisotropic phonon-polaritonic Bloch modes, but also paves the way for their practical application in nanophotonics.

## Abstract (web summary)

This work demonstrates actively controlled, low-loss phonon-polaritonic Bloch modes in a graphene-gated α-MoO_3_ polaritonic crystal, which enables enhanced near-field resonances and switchable far-field leakage through band structure modulation.

## Graphical abstract

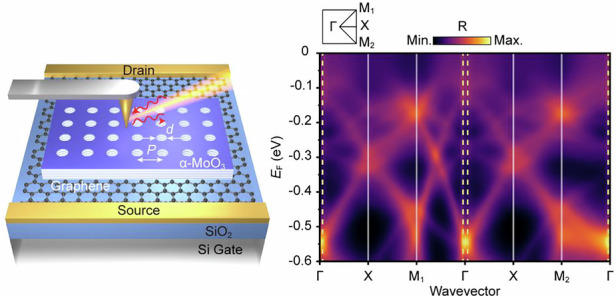

## Introduction

Polaritons—hybrid quasiparticles formed by the strong coupling of light and matter—have enabled unprecedented control and confinement of light at the nanoscale, paving the way for a new generation of compact, high-performance photonic technologies^1–12^. Compared to bulk polaritonic crystals (e.g., calcite^13^, CdWO_4_ (ref. ^14^)), van der Waals materials such as alpha-phase molybdenum trioxide (α-MoO_3_)^15–18^, hexagonal boron nitride^19,20^, and graphene^21,22^ can support low-loss polaritons with tunability and ultra-strong field confinement^1,23–27^. By patterning these materials with subwavelength periodicity, polaritonic crystals (PoCs) significantly enhance light-matter interactions^28–31^, enabling Bragg resonances and precise manipulation of polaritons at the nanoscale^32–43^. These advances have opened new possibilities for high-resolution imaging, molecular sensing, and tunable, high-performance polaritonic devices^35,36^.

Unlike conventional PoCs that utilize isotropic materials and symmetric geometries, emerging anisotropic phonon PoCs, like α-MoO_3_-based systems, enable low-symmetry Bloch modes with high-Q resonances and asymmetric dispersion bands along high-symmetry paths^38^, supporting directional light propagation and energy confinement. Nevertheless, a critical challenge remains: existing anisotropic phonon PoCs are designed with static configurations, rendering their optical properties and band structures fixed after fabrication^38–41^. This static nature hinders their application in adaptive platforms, which demand dynamic reconfigurability and multifunctionality. While isotropic plasmon PoCs based on graphene have showcased dynamic control through electrostatic gating, their practical application is limited by significant plasmonic dissipation^33,35^. To overcome these limitations, we integrate α-MoO_3_ patterned into a periodic hole array with a graphene electrostatic gate, combining the low-loss and anisotropic responses of α-MoO_3_ phonon PoCs with the dynamic tunability of graphene. This hybrid approach enables electrostatic modulation of phonon polaritons (PhPs) in α-MoO_3_ PoCs, tuning losses and iso-frequency contours (IFCs)^26,44,45^. Such tunability achieves precise control over low-loss anisotropic phonon PoCs and aligns with the growing demand for adaptive optical technologies.

In this work, we demonstrate in situ dynamic tuning of Bloch modes in α-MoO_3_ PoCs by integrating them with electrically gated graphene. Rigorous theoretical analysis reveals that a periodic hole array in α-MoO_3_ coupled with a graphene gate supports Bloch modes spanning hyperbolic, canalized, and elliptic dispersion regimes. In the following real-space nano-imaging experiment, it is revealed that tuning the graphene Fermi level modulates Bloch mode intensity, wavelength, and spatial distribution. Furthermore, electrostatic gating actively reshapes the band structure of PoCs, locating the flat-band regions—characterized by zero group velocity—at desired excitation wavelengths, to leverage their intrinsically high density of states (DOS). Interestingly, by strategically shifting flat-band regions in and out of the light cone through gating, we achieve switchable far-field leakage, enabling efficient coupling of Bloch modes to free-space radiation. This mechanism is intuitively corroborated by the gate-dependent Bloch mode distribution in momentum space. Our findings establish a robust framework for precise electrostatic control of anisotropic phonon PoCs, paving the way toward high-performance, adaptive nanophotonic devices.

## Results

### Dynamic tuning of nanoscale Bloch modes

**Fig. 1:**
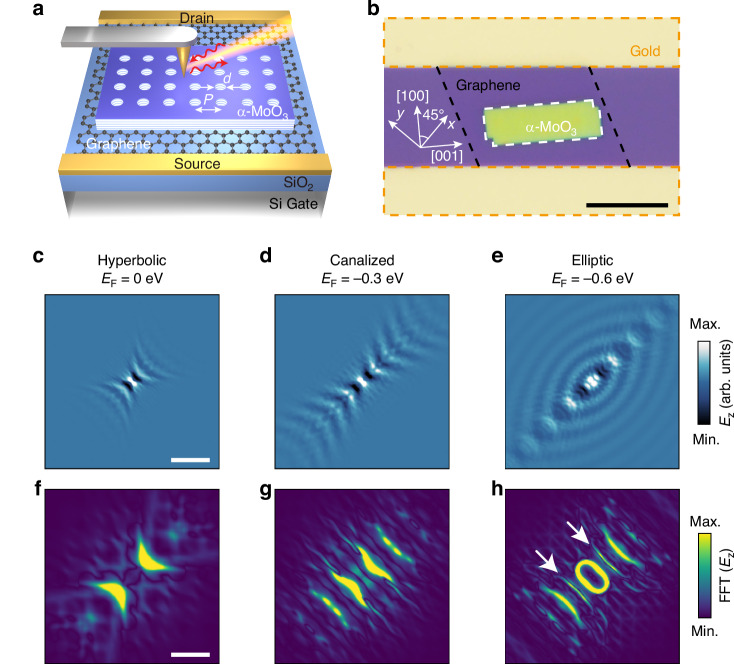
Dynamic tuning of Bloch modes in the α-MoO_3_ PoC/graphene device. **a** Schematic of an α-MoO_3_ PoC/graphene device, consisting of a square periodically perforated α-MoO_3_/graphene heterostructure on a SiO_2_ (285 nm)/Si substrate. The lattice periodicity *P* = 300 nm, with the hole diameter *d* = 50 nm. **b** Optical image of the α-MoO_3_ PoC/graphene device with α-MoO_3_ thickness *t* = 81 nm. The *x*-axis and *y*-axis represent the arrangement directions of the square hole array. The [100] crystallographic axis of α-MoO_3_ forms a 45° angle with respect to the *x*-axis. The white, black, and yellow dashed regions indicate the α-MoO_3_ PoC, graphene, and gold electrodes, respectively. Scale bar: 20 μm. **c**–**e** Calculated electric field distributions of nanoscale Bloch modes in the α-MoO_3_ PoC by a dipole source at *E*_F_ = 0 eV, −0.3 eV, and −0.6 eV. Scale bar: 600 nm. **f**–**h** Corresponding FFT of the electric field distributions in (**c**–**e**). The white arrows indicate the emergent momentum-space feature. Scale bar: 100*k*_0_

Figure 1a, b presents the schematic and optical image of our tunable α-MoO_3_ PoC/graphene device, respectively. The device is fabricated by vertically stacking α-MoO_3_ PoC and graphene on a SiO_2_ (285 nm)/Si substrate (see Materials and Methods). A periodic array of circular air holes, with a diameter *d* = 50 nm and a periodicity *P* = 300 nm, is etched into the α-MoO_3_ layer with a thickness *t* = 81 nm, forming the PoC structure. Since α-MoO_3_ supports anisotropic polaritons, the orientation of the periodic hole lattice critically influences the properties of PoCs. To enable a systematic study of electrostatic tuning, we adopt a high-symmetry configuration by orienting the hole array at *θ* = 45° with respect to the [100] crystallographic axis of α-MoO_3_ (inset in Fig. 1b).

The perforated α-MoO_3_ PoC introduces Bragg scattering, which fundamentally alters polariton propagation, giving rise to nanoscale Bloch modes. To calculate the electric field distributions of Bloch modes, we employ the rigorous coupled-wave analysis (RCWA) and place a dipole source above the α-MoO_3_ PoC at *ω* = 931 cm^−1^ (see section S1.1). Figure 1c–e reveals striking gate-dependent transitions of Bloch modes across hyperbolic (Fig. 1c), canalized (Fig. 1d), and elliptic (Fig. 1e) polaritonic regimes as the graphene Fermi energy *E*_F_ is tuned. The large electrostatic tuning originates from the coupling and hybridization between the hyperbolic PhPs in α-MoO_3_ and the gate-tunable surface plasmon polaritons in graphene, forming hybrid phonon-plasmon polaritons (HPPPs)^44^. Meanwhile, the Bloch modes exhibit pronounced anisotropic propagation, with direction-dependent field enhancement. In particular, at higher *E*_F_ (Fig. 1e), the Bloch modes display enhanced field intensity and more abundant fringes along the [100] crystallographic axis of α-MoO_3_. This gate-dependent evolution reflects two key electrostatic effects: first, increasing the graphene Fermi level *E*_F_ lengthens the polariton wavelength^44^, thereby boosting its overlap and coupling with the periodic hole lattice. Second, the amplified coupling efficiency at higher *E*_F_ strengthens periodic modulation of polaritons, driving a more pronounced redistribution of Bloch modes.

These electrostatic effects are both quantitatively supported by momentum-space analysis via the fast Fourier transforms (FFTs) of the electric field distributions (Fig. 1f–h). In our system, due to the strong field confinement of HPPPs and the perturbative nature of the subwavelength hole array, the wavevector of the dominant Bloch harmonic *k* can be well approximated by *k* + n*G* ≈ *k*_HPPPs_, where *k*_HPPPs_ is the wavevector of HPPPs, n is an integer, and *G* is the reciprocal vector. This relationship is further corroborated by the FFT results, which show that the momentum of the strongest Bloch harmonic closely matches that of the HPPPs. As the Fermi level increases, the inter-hole interactions strengthen, leading to enhanced amplitudes of other harmonic components. These correspond to the additional branches between first- and second-order polariton dispersions observed at higher *E*_F_ (white arrows in Fig. 1h), which sharply contrasts with the momentum-space feature observed in pristine α-MoO_3_ (Fig. S4). Therefore, these calculations in Fig. 1c–h indicate that electrostatic gating enables in situ reconfiguration of anisotropic Bloch modes across distinct polaritonic regimes, while simultaneously modulating their intensity, wavelength, and spatial distribution.

### Real-space nano-imaging of tunable Bloch modes

To probe the nanoscale evolution of Bloch modes under electrostatic control, we employ infrared scattering-type scanning near-field optical microscopy (IR s-SNOM) at *ω* = 931 cm^−1^. In this setup, a gold-coated atomic force microscopy (AFM) tip scans the surface of the α-MoO_3_ PoC, serving as both a launcher and a detector of polaritons. Importantly, the detected signal arises not from direct scattering off the holes, but from interference between tip-excited Bloch modes and their backscattered components modulated by the PoC lattice^34,36^. This interferometric contrast mechanism enables real-space imaging of polariton propagation on the 2D surface with nanoscale spatial resolution well beyond the conventional diffraction limits.

**Fig. 2:**
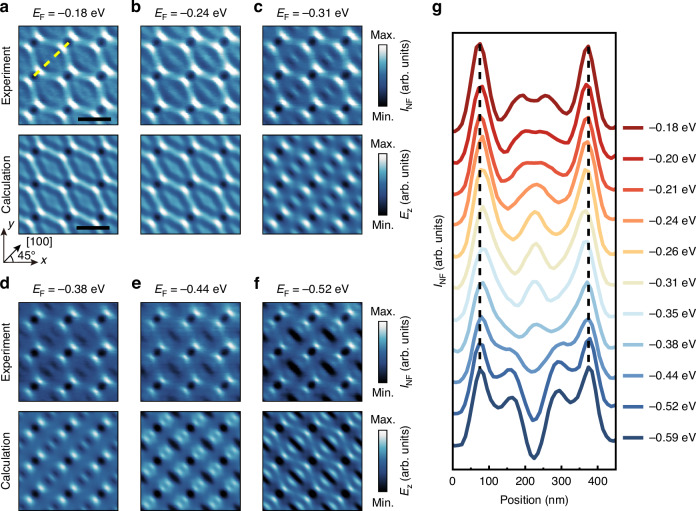
Real-space nano-imaging of Bloch modes in the α-MoO_3_ PoC/graphene device. **a**–**f** Experimental near-field intensity (*I*_NF_, top panels) and corresponding calculated electric field distribution (*E*_z_, bottom panels) of Bloch modes in the α-MoO_3_ PoC/graphene device for *E*_F_ of −0.18 eV (**a**), −0.24 eV (**b**), −0.31 eV (**c**), −0.38 eV (**d**), −0.44 eV (**e**) and −0.52 eV (**f**). Scale bars: 300 nm. **g** Line profiles of Bloch modes extracted along the yellow dashed line in (**a**), with *E*_F_ ranging from −0.18 eV to −0.59 eV. The black dashed lines indicate two peaks of near-field intensity near the holes. The [100] axis is oriented at an angle of 45° relative to the *x*-axis

Figure 2a–f display near-field nano-images of gate-tunable Bloch modes in the α-MoO_3_/graphene PoC device, captured in situ during a back-gate voltage sweep from 130 V to −120 V. These images reveal Bloch modes across the *E*_F_ range from −0.18 eV to −0.59 eV, with the complete set in Fig. S5. The *E*_F_ is determined by gate-dependent third-harmonic generation (THG) measurements^44,46^ (see Section S2). At lower *E*_F_ (Fig. 2a), hyperbolic polariton interference generates Bloch modes with a closed diamond-shaped field pattern, featuring an internal double-fringe structure. Further gate tuning toward medium *E*_F_ induces geometric reconstruction of Bloch modes (Fig. 2b–d), evolving the pattern into discontinuous fringes aligned along the [100] axis. This alignment, where polaritons become collimated along this crystallographic direction, reflects the canalization transition. At higher *E*_F_ (Fig. 2e, f), the polaritons exhibit an elliptical shape, and their Bloch modes show pronounced signal loss (dark regions, not within the hole array) with reduced overall intensity. Notably, the experimental nano-images exhibit excellent agreement with theoretical calculations across all three dispersion regimes (hyperbolic, canalized, elliptic), as shown in Fig. 2a–f. Further evidence of this consistency is provided in Fig. S6, showing a high degree of similarity between the FFT patterns of the experimental and calculated nano-images.

To quantitatively evaluate the electrostatic modulation of Bloch modes, we extract line profiles along the [100] axis between adjacent holes, as illustrated by the yellow dashed line in Fig. 2a. A more comprehensive set of these line profiles, derived from Fig. S5 with a wider range of *E*_F_ values, is presented in Fig. 2g. Within each line profile, two peaks consistently occur near the holes across all *E*_F_ values. These peaks, marked by the black dashed lines, correspond to constructive interference, forming bright spots at the hole boundaries in the nano-images. At lower *E*_F_, the region between the peaks appears relatively flat, but with increasing *E*_F_, a distinct additional peak emerges, ultimately forming a trough at higher *E*_F_. Such dynamic evolution is characterized by the directional migration of the fringes along the [100] axis with increasing *E*_F_. This mirrors the changing intensity patterns observed in the nano-images between the holes: transitioning from uniform at lower *E*_F_, to bright fringes at increased *E*_F_, and finally to dark regions at higher *E*_F_. From these observations, we infer that the variation in polariton dispersion induced by the gate directly alters the wavelengths of the Bloch modes. This, in turn, modifies the spacing of the interference fringes and drives a systematic redistribution of the near-field signal.

Complementary measurements at θ = 0° (see Fig. S7) confirm the gate-driven reconfiguration of directional Bloch modes interference fringes along the [100] axis, showing similar peak migration, central coalescence, and intensity modulation patterns, even with altered phonon PoC symmetry. This similar behavior under rotational perturbation highlights the robustness of electrostatic gate control over polaritonic Bloch modes across different crystal orientations. The reduced resolution of fringe detail in experimental nano-images is likely due to intrinsic loss mechanisms, such as material inhomogeneities or fabrication imperfections, which are not fully accounted for in theoretical calculations.

### Dynamic control of the band structure

**Fig. 3:**
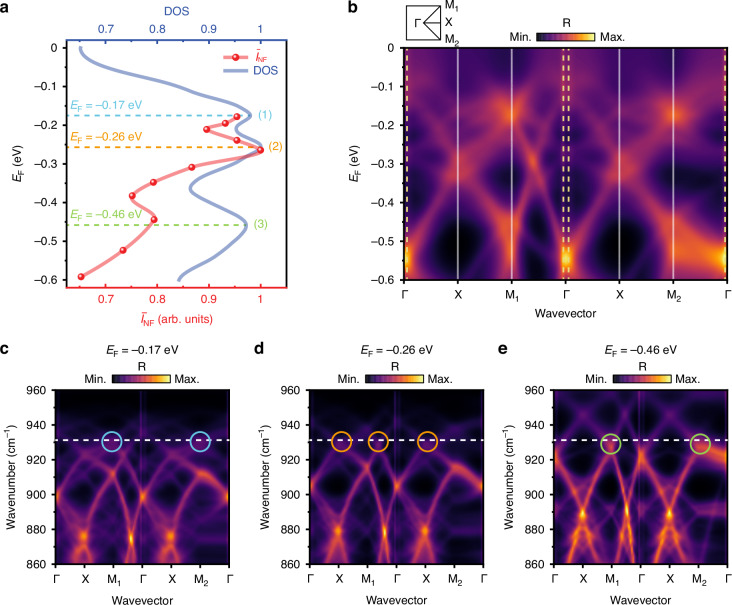
Dynamic reshaping of Bloch mode band structure. **a** Integrated near-field intensity spectrum (red dots) across the whole unit cell and theoretically calculated DOS (blue curve) via integration over the first Brillouin zone. The red curve is a guide for the eye. The blue, orange, and green dashed lines mark three resonant peaks. **b** Theoretically calculated band structure of the α-MoO_3_ PoC as a function of *E*_F_ at a fixed frequency of 931 cm^−1^. The yellow dashed lines indicate the free space light cone. Inset: the first Brillouin zone of the square-type PoC. **c**–**e** Theoretically calculated band structures of the α-MoO_3_ PoC at a fixed *E*_F_ of −0.17 eV (**c**), −0.26 eV (**d**), and −0.46 eV (**e**), respectively. The white dashed lines mark the experimental IR laser frequency *ω* = 931 cm^−1^, while the blue, orange, and green circles indicate the corresponding flat-band regions

We now focus on the dynamic control of the Bloch mode band structure, a crucial aspect for comprehending the modulation mechanism. This exploration involved spatially integrating near-field signals across the entire unit cell of the α-MoO_3_ PoC, thereby producing a near-field intensity spectrum where the near-field intensity is a function of *E*_F_ (Fig. 3a, red curve). The experimental spectrum exhibits three resonant peaks at *E*_F_ = −0.17 eV, −0.26 eV, and −0.46 eV, coinciding with the peaks in the calculated DOS (blue curve). This spectral alignment directly links gate-modulated near-field response to the band structure characteristics.

These spectral features originate from the electrostatically driven band structure, as evidenced by the momentum-space analysis along the high-symmetry path in the first Brillouin zone (Fig. 3b). This gate-dependent band structure is calculated as a function of *E*_F_ at *ω* = 931 cm^−1^ using RCWA (see section S1.2). Notably, the intrinsic anisotropy of α-MoO_3_ breaks the four-fold rotational symmetry (C_4_) of the square-lattice structure. With the [100] crystal axis oriented at *θ* = 45°, this symmetry breaking manifests as non-degenerate band structures along the Γ–X–M_1_–Γ and Γ–X–M_2_–Γ paths. Each resonant peak in Fig. 3a coincides with a flat-band region on the dispersion branches in Fig. 3b—where the group velocity vanishes—analogous to van Hove singularities in conventional band structures of photonic or polaritonic crystals^36,47^. Such flat-band regions lead to a significant increase in DOS, inducing a pronounced near-field enhancement.

To connect these flat-band-induced resonances to the band structure evolution, we calculate the band structures at three representative *E*_F_ values in Fig. 3c–e, corresponding to the resonances observed in Fig. 3a. The presence of a flat band is identified by the splitting of originally degenerate points induced by the nanohole array, where the band dispersion on either side exhibits opposite group velocities, resulting in a near-zero net group velocity in the vicinity, i.e., flat-band regions. As *E*_F_ increases, the band structure undergoes a continuous blue shift. This shift strategically brings different flat-band regions into coincidence with the fixed excitation frequency, enabling selective enhancement of the DOS at specific *E*_F_. The alignment between the excitation frequency (*ω* = 931 cm^−1^) and the flat-band regions is marked by blue (*E*_F_ = −0.17 eV), orange (*E*_F_ = −0.26 eV), and green (*E*_F_ = −0.46 eV) circles in Fig. 3c–e, confirming that the resonant peaks in Fig. 3a originate from this gate-controlled spectral alignment. The overall evolution of the band structure is further validated by its consistency with finite-difference time-domain (FDTD) simulations (Section S3, Fig. S3).

Interestingly, the flat-band regions around *E*_F_ = −0.55 eV in the band structure do not produce a detectable resonance in Fig. 3a. We attribute this absence to the enhanced far-field leakage of Bloch modes at elevated *E*_F_, as quantitatively demonstrated in Section S4. Our results in Fig. S9 reveal a pronounced enhancement in far-field radiation at *E*_F_ = −0.55 eV, indicating that the Bloch resonance at this Fermi level efficiently couples energy to free-space radiation. Specifically, low-momentum Bloch modes within the light cone around the Γ-point (yellow dashed lines in Fig. 3b) radiate into free space, thereby attenuating their near-field contribution. This mechanism also accounts for the overall intensity reduction observed at higher *E*_F_ in Fig. 2f. Importantly, the above demonstration of tuning far-field leakage via electrostatic gating introduces a novel pathway for engineering reconfigurable nanophotonic cavities with dynamically switchable radiative coupling.

### Bloch mode distribution in momentum space

**Fig. 4:**
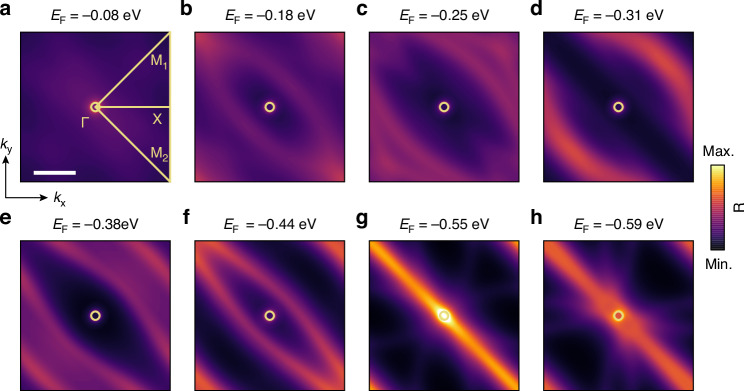
The Bloch mode distributions within the first Brillouin zone. **a**–**h** Calculated IFCs in the first Brillouin zone for the α-MoO_3_ PoC/graphene device at a fixed frequency of 931 cm^−1^, demonstrating gate-dependent dispersion for *E*_F_ of −0.08 eV (**a**), −0.18 eV (**b**), −0.25 eV (**c**), −0.31 eV (**d**), −0.38 eV (**e**), −0.44 eV (**f**), −0.55 eV (**g**) and −0.59 eV (**h**). The yellow circles indicate the light cone. Scale bar: 10*k*_0_

Building upon our previous discussion, we now present a more holistic view of the Bloch mode distribution in momentum space. Our specific focus is on the evolution of IFCs (*ω* = 931 cm^−1^) across the first Brillouin zone (Fig. 4a–h), which offers insights into the manipulation of phonon-polaritonic Bloch modes through electrostatic gating. Notably, the IFCs of the Bloch modes evolve non-uniformly under gating—some expanding outward while others converging inward. This behavior originates from the relative positions and movements of the folded dominant Bloch modes derived from the two underlying HPPP branches. As *E*_F_ increases, the Bloch modes derived from the *k*_100_ < 0 branch shift rightward along the Γ–M_1_ direction, while the modes from the *k*_100_ > 0 branch shift leftward. Convergence occurs when such a pair of modes moves toward each other, while expansion occurs when they cross and then separate. To further elucidate this mechanism, we calculate the unfolded and folded HPPPs’ IFCs at *E*_F_ = −0.25 eV. Since the wavevector of the dominant harmonic of the Bloch modes closely matches that of the underlying HPPPs, the trends observed in the HPPPs’ IFCs clearly reflect those of the Bloch modes. As shown in Fig. S10, the calculated folded IFCs of the HPPPs exhibit the same simultaneous expansion and convergence behavior, which provides strong visual evidence for the physical origin of the gate-tunable Bloch mode dynamics. Despite this non-uniform behavior, the evolution consistently occurs along the Γ–M_1_ direction in momentum space, which explains the migration of interference fringes along the [100] crystallographic axis in real space (Fig. 2). The study of IFC evolution also uncovers gate-controlled far-field leakage channels. Bloch modes maintain their near-field confinement for |*E*_F_| below a critical threshold of 0.55 eV, as demonstrated by their momentum-space localization outside the free-space light cone (yellow circles in Fig. 4a–f). However, beyond this threshold, partial modes at flat-band regions breach the light cone, thereby enabling efficient coupling to far-field radiation (Fig. 4g, h). These findings highlight the role of electrostatic gating in PoCs as a dynamic tool for steering Bloch mode propagation through momentum-space dispersion engineering, especially in terms of switching far-field leakage via light-cone engineering.

## Discussion

In summary, we have established a novel platform for dynamic control of anisotropic phonon-polaritonic Bloch modes in α-MoO_3_ PoC/graphene heterostructures. By combining the electrical tunability of graphene plasmons with the intrinsically low-loss and strongly anisotropic PhPs in α-MoO_3_, this hybrid system enables unprecedented in situ manipulation of Bloch modes at the nanoscale. Through experimental nano-imaging and rigorous theoretical analysis, we demonstrate comprehensive gate-dependent modulation of Bloch mode intensity, wavelength, and spatial distribution. Crucially, electrostatic gating enables the dynamic positioning of flat-band regions, which allows us to exploit their inherently strong DOS at the desired wavelength. Furthermore, we achieve dynamic, switchable far-field leakage by strategically tuning the flat-band regions relative to the light cone. This tuning, visualized through gate-dependent Bloch mode distribution in momentum-space, provides direct evidence of the directional evolution of near-field distribution, corroborating this switching mechanism.

This platform exhibits powerful versatility and significant advantages over static PoCs. Unlike systems requiring physical reconfiguration, our approach achieves dynamic control purely through electrical means, leveraging the complementary strengths of graphene and α-MoO_3_. While this tunability could be further optimized for practical applications by implementing more efficient gating strategies, such as high-κ dielectric layers or ionic gates^48,49^, our platform offers compelling benefits for integration into ultra-compact photonic devices. The dynamic tunability, directional modulation capability, and switchable far-field coupling demonstrated here suggest promising potential for applications such as electrically programmable metasurfaces and on-chip beam steering. These advancements significantly broaden the applicability of dynamically tunable phonon-polaritonic PoCs in practical nanophotonics, paving the way for next-generation adaptive devices.

## Materials and Methods

### Sample and device fabrication

To fabricate the α-MoO_3_ PoCs, α-MoO_3_ flakes were initially obtained through mechanical exfoliation from bulk crystals (SixCarbon Technology, Shenzhen). These flakes were deposited onto 285 nm SiO_2_/Si substrates that had been treated with oxygen plasma (SUNJUNE PLASMA VP-R3). The fabrication of perforated α-MoO_3_ flakes followed, employing electron beam lithography (EBL) and reactive ion etching (RIE).

For the construction of the α-MoO_3_ PoCs/graphene devices, a monolayer of graphene, exfoliated from graphite crystals (Shanghai Onway Technology Co., Ltd.), was placed onto an oxygen plasma-treated 285 nm SiO_2_/Si substrate. Drain and source electrodes, comprising 5 nm Cr and 50 nm Au, were deposited through thermal evaporation. The appropriately thick α-MoO_3_ flake was carefully transferred onto the graphene using a micromanipulator. Finally, the entire sample was mounted onto a compact chip carrier, with all electrodes wire-bonded to enable electrical gating and doping.

### Near-field characterization

Near-field characterization was carried out using a commercially available IR s-SNOM system (Bruker nanoIR3s), which operates based on a tapping mode AFM setup. This system is integrated with a CO_2_ laser (Access Laser, L4SL-13CO2), which can generate p-polarized IR beam with a frequency of 931 cm^−1^. The IR beam is focused on the apex of a gold-coated AFM tip oscillating at ∼220 kHz. The back-scattered light from the tip is collected by an off-axis parabolic mirror and directed towards a HgCdTe (MCT) photodetector. The near-field signal is demodulated at the second harmonic of the tip oscillation frequency to properly suppress background signals. The integrated near-field intensity spectrum shown in Fig. 3a is then obtained by spatially integrating the near-field amplitude signal *I*_NF_ across the entire unit cell of the α-MoO_3_ PoC.

### Electrostatic gating

Both the near-field and THG measurements were conducted while sweeping the back-gate voltage, which was applied using a SourceMeter (Keithley, 2450), and the graphene resistance was simultaneously recorded using a lock-in amplifier (Model OE1022, Guangzhou Sine Scientific Instrument Co., LTD). The α-MoO_3_ PoC/graphene device was maintained in a dry air environment at room temperature throughout the measurements.

## Supplementary information

Supplementary Information for Dynamic tuning of Bloch modes in anisotropic phonon polaritonic crystals

## Data Availability

All data needed to evaluate the conclusions in this study are presented in the main text and in the supplementary information. The raw data generated in this study are available from the corresponding authors upon reasonable request.
