# Supplementary material for: Dynamic tuning of Bloch modes in anisotropic phonon polaritonic crystals
Source: Light Sci Appl. 2026 Jan 3;15:41. doi: 10.1038/s41377-025-02157-6 (PMC12764765; doi:10.1038/s41377-025-02157-6)
Supplement: Supplementary file 1 — Supplementary Information for Dynamic tuning of Bloch modes in anisotropic phonon polaritonic crystals [file 41377_2025_2157_MOESM1_ESM.docx]

**Supplementary Information for**

**Dynamic tuning of Bloch modes in anisotropic phonon polaritonic crystals**

Junbo Xu,^1^† Ke Yu,^1^† Xiang Ni,^2^† Enrico M. Renzi,^3,4^ Lei Zhou,^1^ Yanzhen Yin,^1^ Zhou Zhou,^1^ Zhichen Zhao,^1^ Tao He,^1^ Di Huang,^1^ Kyoung-Duck Park,^5^ Zhanshan Wang,^1,6^ Andrea Alù,^3,4^* and Tao Jiang^1,6^*

^1^MOE Key Laboratory of Advanced Micro-Structured Materials, Shanghai Frontiers Science Center of Digital Optics, Institute of Precision Optical Engineering, and School of Physics Science and Engineering, Tongji University, Shanghai 200092, China.

^2^School of Physics, Central South University, Changsha, Hunan 410083, China.

^3^Photonics Initiative, Advanced Science Research Center, City University of New York, New York, NY 10031, USA.

^4^Physics Program, The Graduate Center, City University of New York, New York, NY 10026, USA.

^5^Department of Physics and Department of Semiconductor Engineering, Pohang University of Science and Technology (POSTECH), Pohang 37673, Republic of Korea.

^6^Shanghai Institute of Intelligent Science and Technology, Tongji University, Shanghai 200092, China.

*Correspondence: Andrea Alù ([aalu@gc.cuny.edu](mailto:aalu@gc.cuny.edu)); Tao Jiang ([tjiang@tongji.edu.cn](mailto:tjiang@tongji.edu.cn))

†These authors contributed equally: Junbo Xu, Ke Yu, Xiang Ni

**Section 1. Theoretical calculations**

The electric field distributions and band structure of Bloch modes in our α-MoO_3_ PoC/graphene device were theoretically calculated using rigorous coupled-wave analysis (RCWA) method^1^. In the theoretical calculation, perforated α-MoO_3_ PoCs were modeled as a structure containing a square periodic array of air holes (Fig. S1), where the permittivity inside the holes was that of air, and outside the holes was that of the material.

**Section 1.1 Theoretical calculation of the electric field distributions**

Considering that the nanostructure of α-MoO_3_ PoCs is much smaller than the IR laser wavelength in the near-field measurements, we employ the quasi-static approximation in the calculation of the electric field distributions. The quasi-static approximation is employed to model the atomic force microscopy (AFM) tip-sample interaction, justified by its deeply subwavelength spatial scale (~20 nm) compared to the infrared excitation wavelength (~10.7 µm). This approximation remains valid regardless​ of the polariton wavelength *λ*_HPPPs_ and Fermi levels, and retardation effects are negligible given the deeply subwavelength character of the tip. We assume AFM tip as a **z** oriented dipole source with a magnitude of *p*, positioned at (***r***_0_, *z*_tip_). The scalar potential distributions of α-MoO_3_ PoCs can be written as follows^2^:

where ***G*** is the reciprocal vector, $\tilde{r}_{G}(k, z)$is the reflection coefficient calculated through RCWA. We only record the field under the dipole source $\Phi_{\text{record}}(r_{0}, z_{\text{record}})$. Thus, the recorded potential field of dipole source located at $(x, y, z_{\text{tip}})$ can be expressed as^2^:

and the electric field satisfies:

$$E=-\nabla\Phi$$

**Section 1.2 Theoretical calculation of band structure**

The RCWA method was used to visualize the band structure of α-MoO_3_ PoCs. We calculate reflection coefficient correspond to the primary Bloch vector ***k*** and the wave vectors of adjacent Brillouin zones ***k***_adj_. Thus, the dispersion visualization can be done by calculating:

$$\sum_{k_{\text{adj}}} \Im\left(\sum_{G} \tilde{r}_{\mathbf{G}}(k_{\text{adj}}, z)\right)+\left(\text{Im}(\sum_{G} \tilde{r}_{G}(k, z))\right)$$

**Section 2. Determination of graphene CNP and *E*_F_**

The determination of graphene CNP was attained by gate-dependent THG intensity measurements, conducted immediately after the near-field optical experiments. For THG measurements, a linearly polarized femtosecond laser beam (Levante IR fs, APE, and Flint, Light Conversion) with a wavelength of 1770 nm (0.70 eV) was focused normally onto the α-MoO_3_ PoCs/graphene device through a 0.5 NA reflective objective (Thorlabs, LMM40X-P01). The back-reflected THG signal was guided to a fiber-coupled spectrometer (Teledyne Princeton Instruments, HRS-300). The THG signal of graphene was gathered simultaneously while sweeping the back-gate voltage $V_{\text{g}}$ from −100 V to 110 V. The gate-dependent THG intensity exhibit a shoulder-like feature (red arrow in Fig. S2) when 2|*E*_F_| approaches the photon energy , where $\omega_{0}$ is the angular frequency of the incident light^3^. From this, we can determinate that the 2*E*_F_ at the $V_{\text{g}}$ marked by red arrows is −0.70 eV, indicating hole doping in graphene.

The relationship between *E*_F_ and $V_{\text{g}}$ is given by^4^:

$$V_{\text{g}}-V_{\text{CNP}}=\frac{E_{\text{F}}}{e}+\frac{ne}{C_{\text{g}}}$$

where $V_{\text{CNP}}$ is the voltage at the graphene CNP, *e* is the electron charge, is the reduced Planck’s constant, $v_{\text{F}}$ is the Fermi velocity in graphene (1.1 × 10^6^ ms^−1^), and $C_{\text{g}}=\varepsilon\varepsilon_{0}/d_{\text{g}}$ is the gate capacitance with $\varepsilon$, $\varepsilon_{0}$, and $d_{\text{g}}$ denote the dielectric constant, the permittivity of vacuum, and the thickness of the gating material (285 nm SiO_2_ in our device), respectively. Thus, we can derive the $V_{\text{CNP}}$ from equations (S7) and (S8). Based on the $V_{\text{CNP}}$, we can further calculate the *E*_F_ at other $V_{\text{g}}$ using equations (S7) and (S8).

**Section 3. Band structure simulations using FDTD**

To verify the accuracy of the theoretical calculations obtained via the RCWA method, we independently computed the band structure using the finite-difference time-domain (FDTD) method (Fig. S3), implemented in the commercially available software Lumerical FDTD (2020b, http://www.lumerical.com/tcad-products/fdtd/). Bloch boundary conditions were applied in the **x** and **y** directions, while perfectly matched layers (PML) were used along the **z** direction. A set of randomly positioned and oriented electric dipoles were placed within a single PoC unit cell to excite Bloch modes, and electric field monitors at random locations were used to capture the excited fields, from which the band structure was extracted.

**Section 4. Gate-dependent** **far-field radiation and absorption losses of the Bloch modes**

To quantitatively validate the mechanism of "switchable far-field leakage", we calculate the far-field radiation of the Bloch mode as a function of *E*_F_ using RCWA. An incident wave with an in-plane momentum of (0 × *G*_x_, 1 × *G*_y_) is used to directly excite the corresponding Bloch mode resonance, enabling the direct probing of its associated loss channels.

Our results, summarized in Fig. S9, reveal a pronounced enhancement in the far-field radiation at *E*_F_ = −0.55 eV. This enhancement indicates a highly efficient energy dissipation mechanism for the Bloch mode at this gating condition, where a significant fraction of the energy is channeled into far-field radiation (leakage). The absorption loss, which is proportional to the real part of graphene's optical conductivity, shows negligible variation with Fermi level in the high-doping regime. Therefore, the diminished DOS is primarily attributed to the intensified far-field leakage. This radiative loss mechanism efficiently depletes the near-field energy, providing a direct explanation for the pronounced signal attenuation observed at the flat-band condition in Fig. 2f of the main text. Our result unequivocally confirms the physical mechanism behind the switchable far-field leakage.**Fig. S1.**


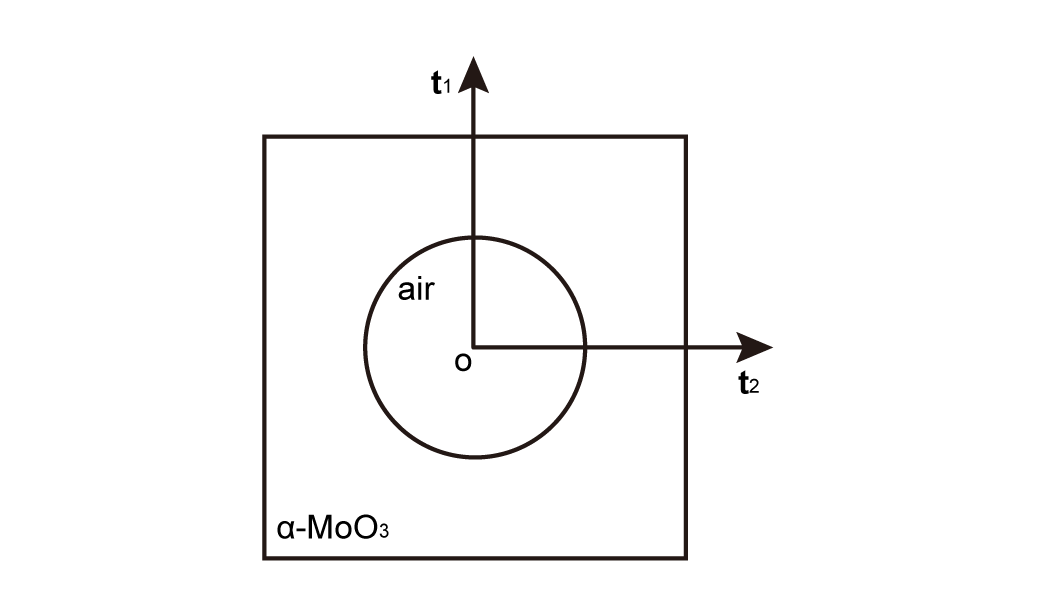


**Fig. S1. Schematic of the structures used in the theoretical calculation.** Unit structure of the periodic square perforated α-MoO_3_ PoCs. Inside the holes is air, while outside the holes is α-MoO_3_ material.

**Fig. S2.**


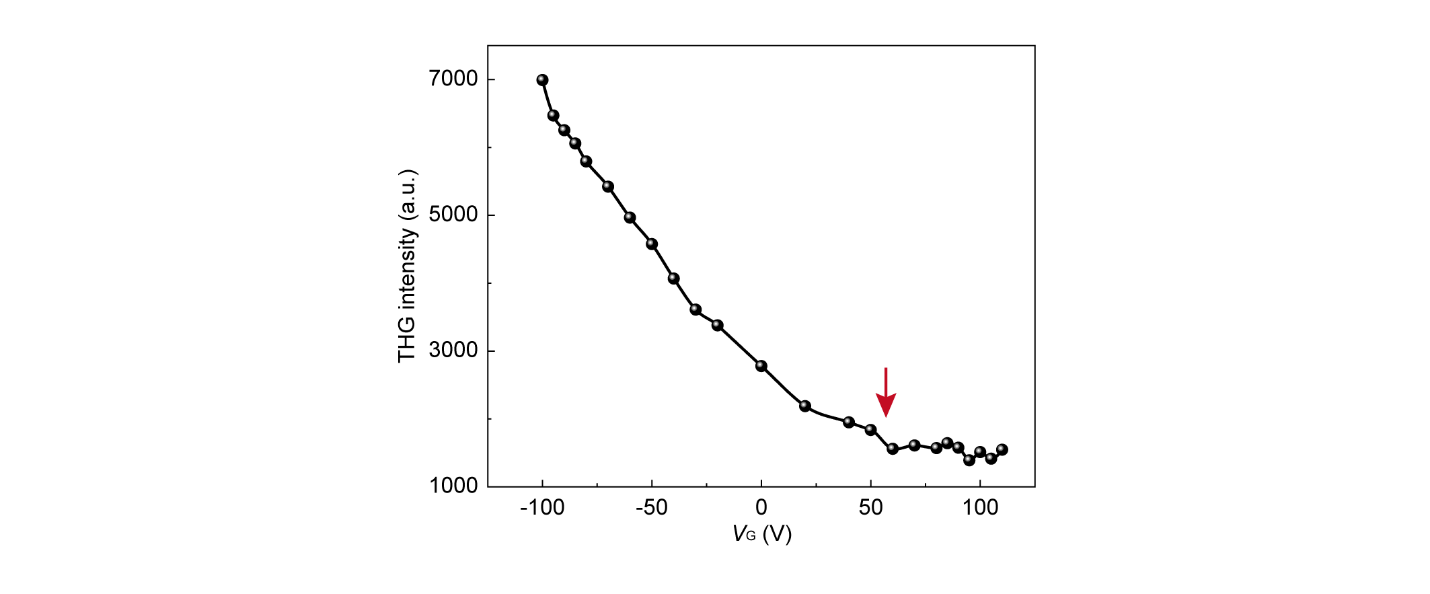


**Fig. S2. Gate-dependent THG.** Gate-dependent THG signal from the monolayer graphene beneath the α-MoO_3_ PoCs, generated by a femtosecond laser beam at 1770 nm (0.70 eV).

**Fig. S3.**


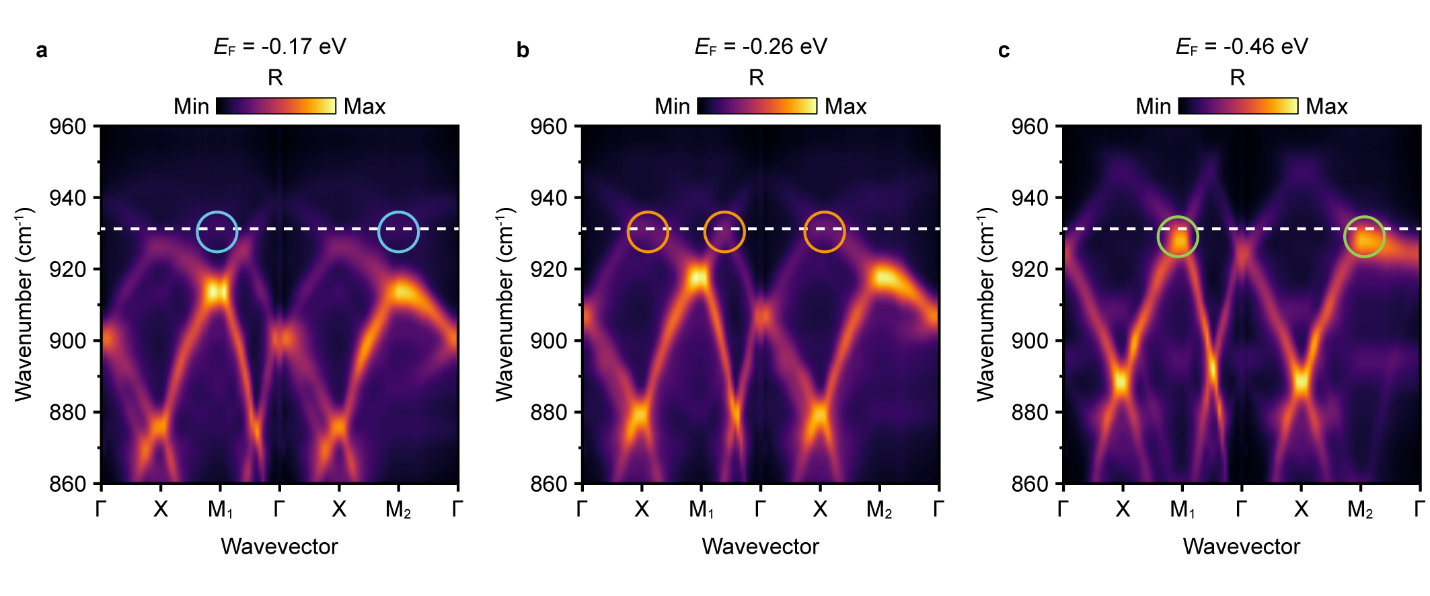


**Fig. S3. Band structures calculated using FDTD method.** **a–c** Band structures of the α-MoO_3_ PoC at a fixed *E*_F_ of −0.17 eV (**a**), −0.26 eV (**b**), and −0.46 eV (**c**), respectively. The white dashed lines mark the experimental IR laser frequency *ω* = 931 cm^−1^. The blue, orange, and green circles indicate the flat-band regions. The [100] axis is oriented at *θ* = 45°.

**Fig. S4.**


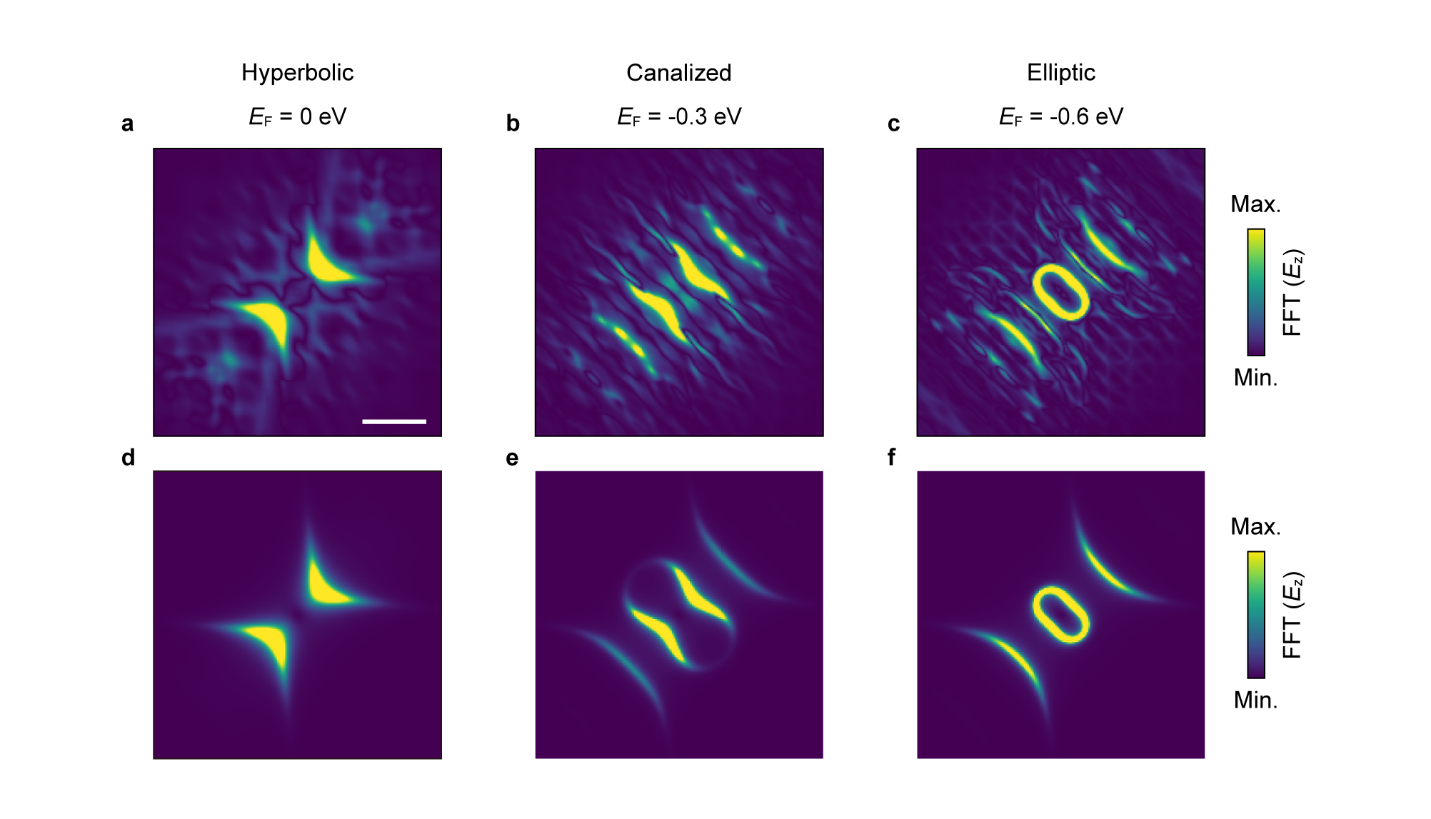


**Fig. S4. Comparison of the FFT of calculated electric field distributions between perforated and pristine α-MoO_3_.** **a–c** FFT of calculated electric field distributions in perforated α-MoO_3_. **d–f** FFT of calculated electric field distributions in pristine α-MoO_3_. Scale bars: 100*k*_0_. The α-MoO_3_ [100] crystallography axis is oriented at *θ* = 45°.

**Fig. S5.**


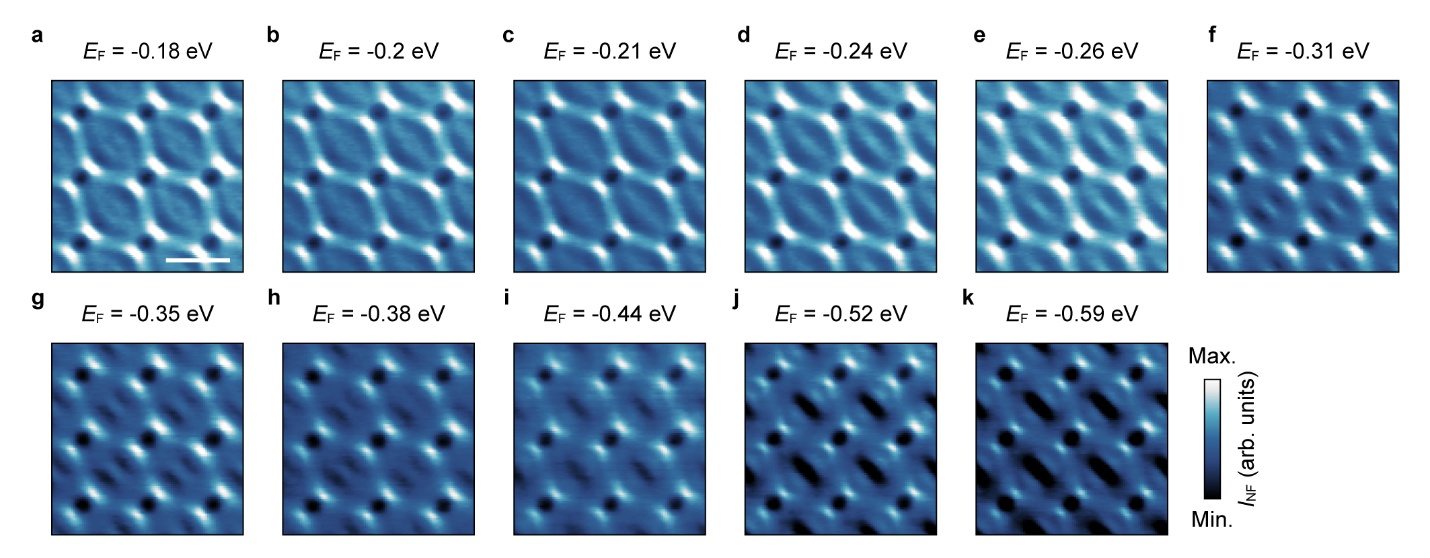


**Fig. S5. Gate-tuning Bloch modes in the α-MoO_3_ PoC/graphene device with a *θ* of 45°.** **a–k** Experimental near-field intensity of HPPPs Bloch modes in the α-MoO_3_ PoC/graphene device for various *E*_F_. Scale bars: 300 nm.

**Fig. S6.**


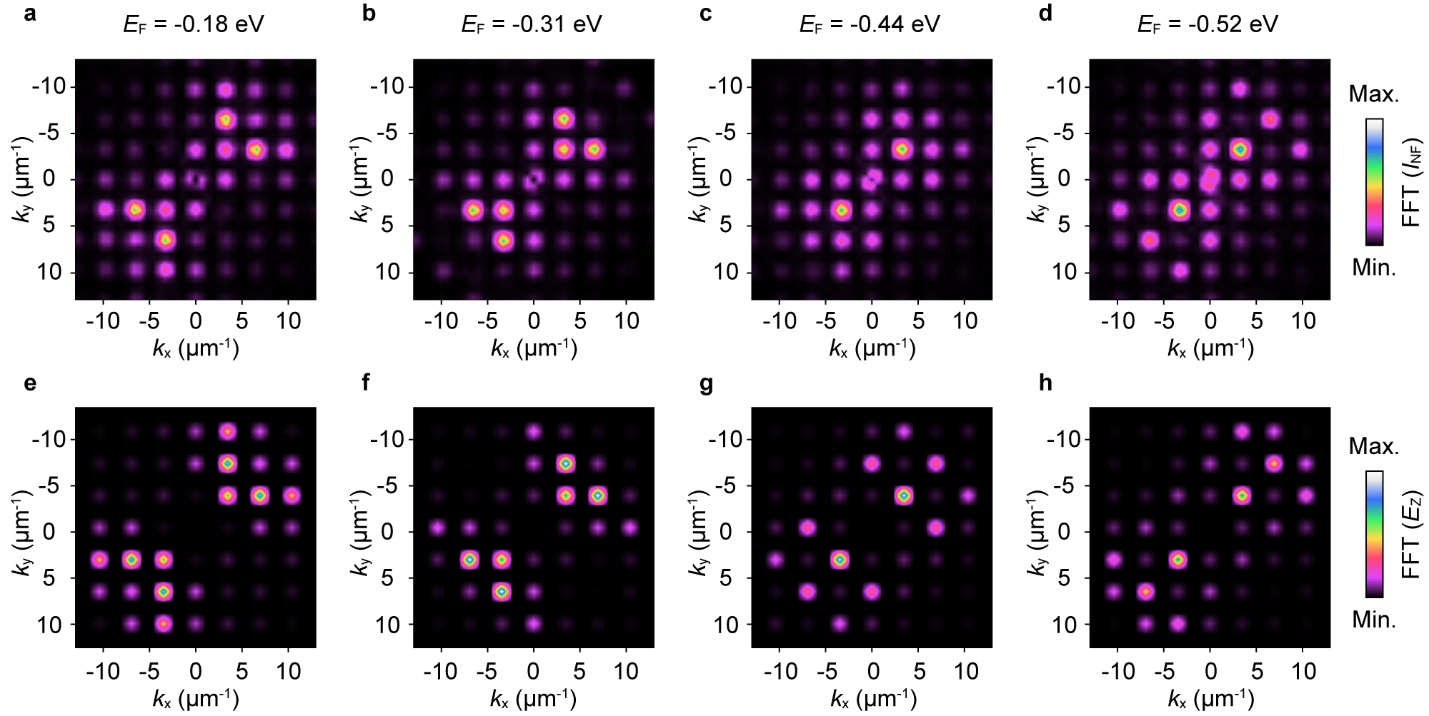


**Fig. S6. Fast Fourier transform (FFT) of experimental and calculated nano-images.** **a–d** FFT of experimental nano-images at *E*_F_ = −0.18 eV (**a**), −0.31 eV (**b**), −0.44 eV (**c**), and −0.52 eV (**d**), respectively. **e–h** FFT of calculated nano-images at *E*_F_ = −0.18 eV (**e**), −0.31 eV (**f**), −0.44 eV (**g**), and −0.52 eV (**h**), respectively.

**Fig. S7.**


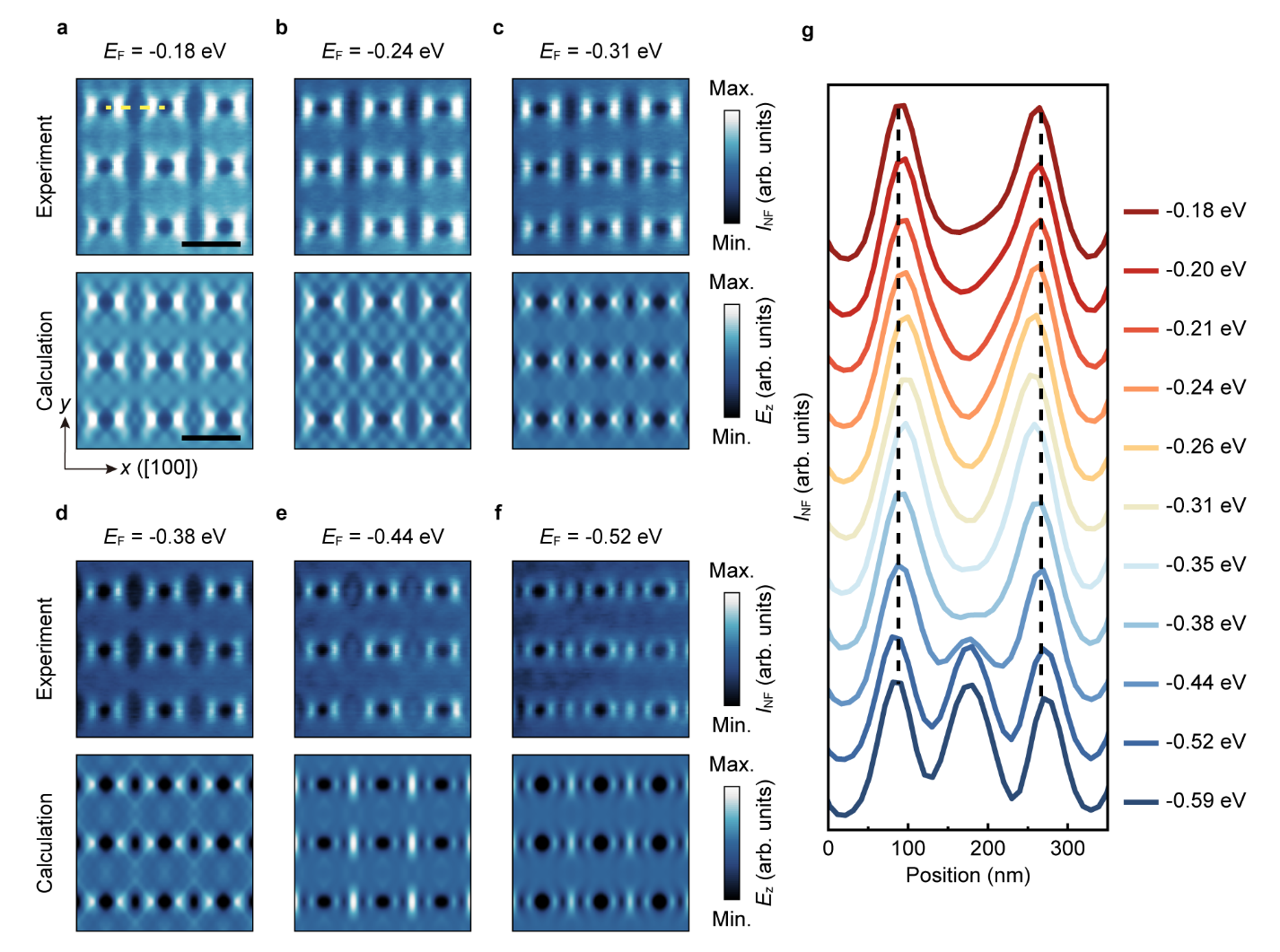


**Fig. S7. Real-space nano-imaging of Bloch modes in the α-MoO_3_ PoC/graphene device with a *θ* of 0°.** **a–f** Experimental near-field intensity (*I*_NF_, top panels) and corresponding calculated electric field distribution (*E*_z_, bottom panels) of Bloch modes in the α-MoO_3_ PoC/graphene device for various *E*_F_. Scale bars: 300 nm. **g** Line profiles of Bloch modes extracted along the yellow dashed line in (**a**), with *E*_F_ ranging from −0.18 eV to −0.59 eV. The black dashed lines indicate two peaks of near-field intensity near the holes.

**Fig. S8.**


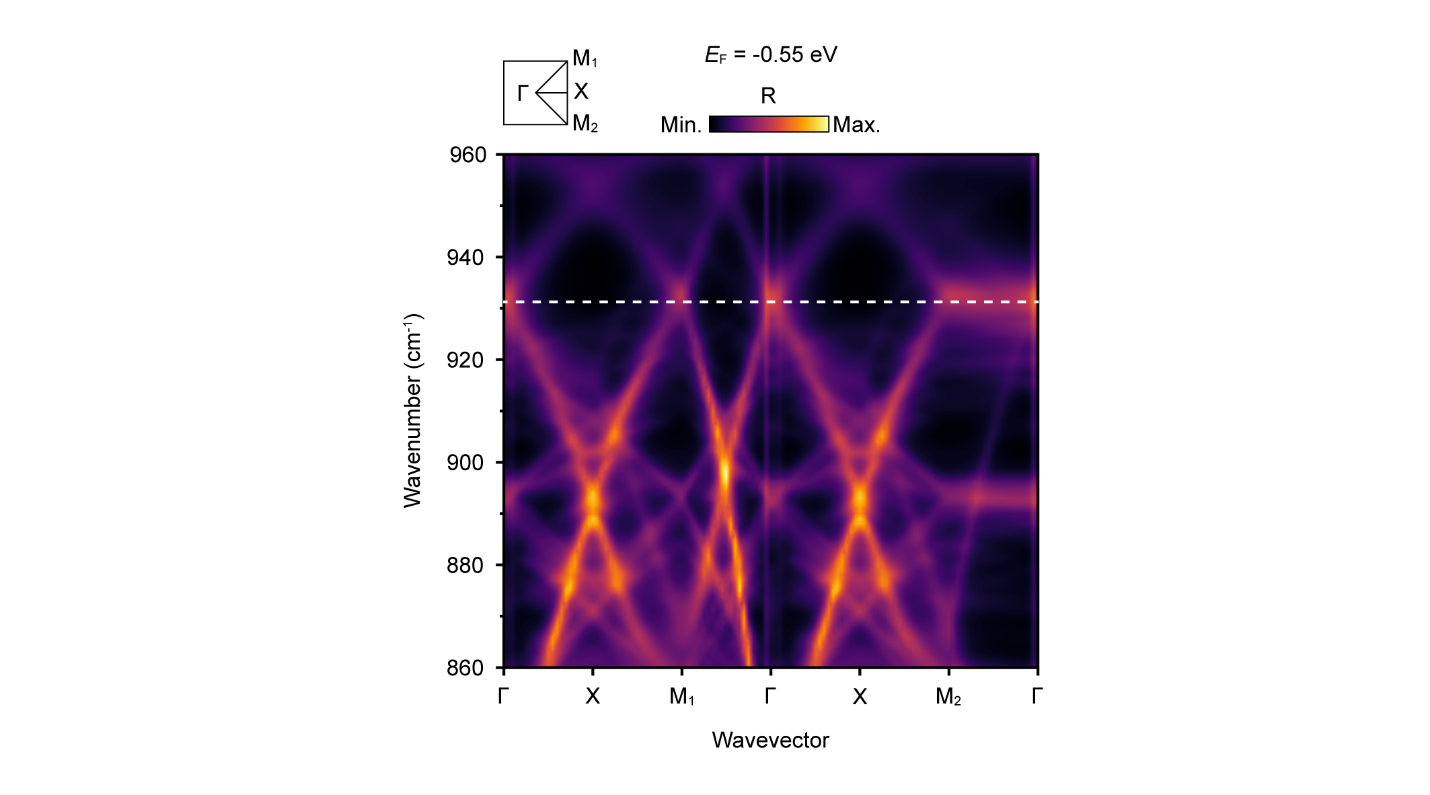


**Fig. S8. Band structure of the α-MoO_3_ PoC at a fixed *E*_F_ = −0.55 eV.** The white dashed lines mark the experimental IR laser frequency *ω* = 931 cm^−1^.

**Fig. S9.**


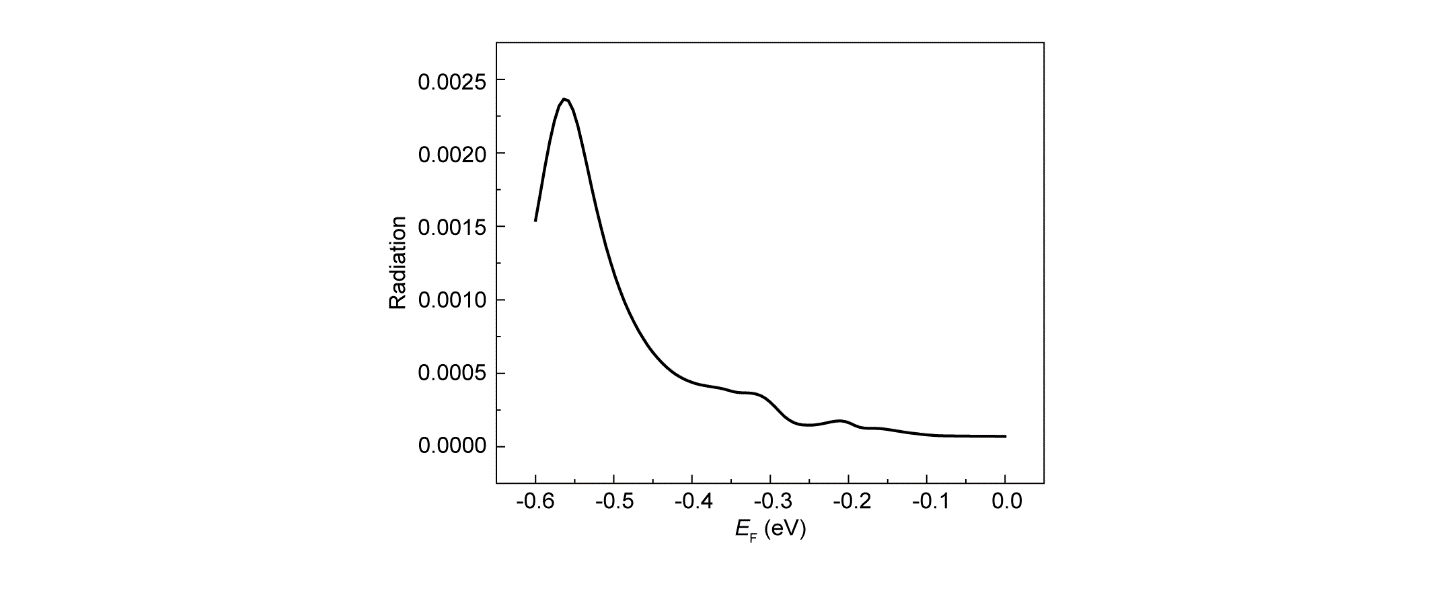


**Fig. S9. Gate-dependent far-field radiation.** The radiation represents the far-field energy flow emitted from the PoCs. An enhancement in far-field radiation is observed near *E*_F_ = −0.55 eV.**Fig. S10.**


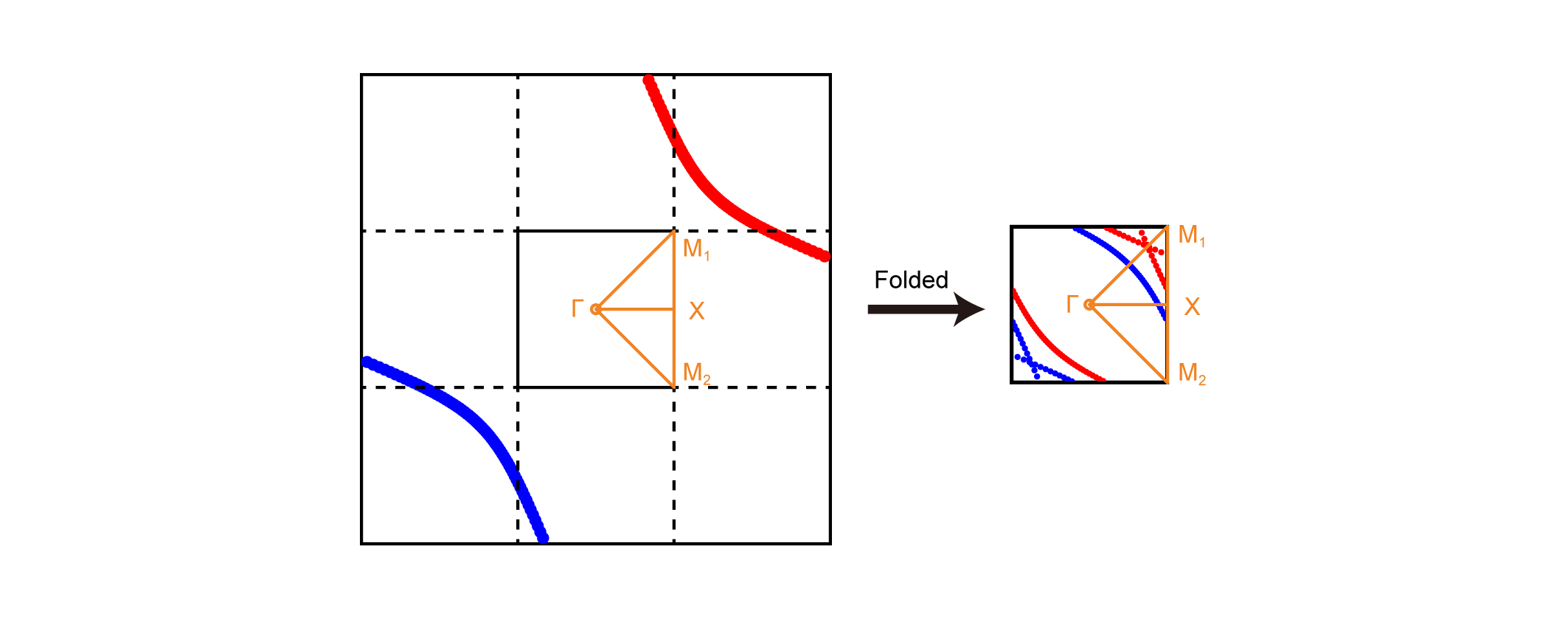


**Fig. S10. Folding process of HPPPs at *E*_F_ = −0.25 eV.** The right branch (red) shifts leftward along Γ**–**M_1_ direction (i.e., [100] crystallographic axis) with increasing ­*E*_F_, while the left branch (blue) shifts rightward. After the folding process, the inner pair of HPPPs expands with *E*­_F_, while the outer pairs converge.

**Fig. S11.**


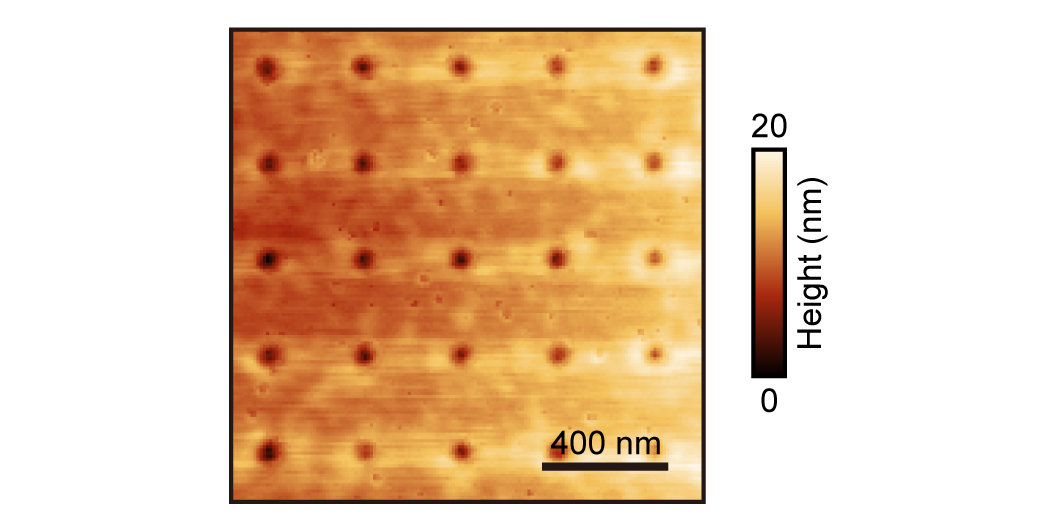


**Fig. S11. AFM image of the PoC in Fig. 1b**. The hole array has a period of 300 nm and a hole diameter of 50 nm. Scale bar: 400 nm.

**References**

1. Rumpf R. C. Improved Formulation of Scattering Matrices for Semi-Analytical Methods That Is Consistent with Convention. *Prog. Electromagn. Res. B* **35**, 241–261 (2011).
2. Yin, Y. *et al.* Selective Excitation of Bloch Modes in Canalized Polaritonic Crystals. *Adv. Opt. Mater.* ***13***, 2403536 (2025).
3. Jiang, T. *et al.* Gate-tunable third-order nonlinear optical response of massless Dirac fermions in graphene. *Nat. Photonics* **12**, 430**–**436 (2018).
4. Das, A. *et al.* Monitoring dopants by Raman scattering in an electrochemically top-gated graphene transistor. *Nat. Nanotechnol.* **3**, 210**–**215 (2008).
